# Spare-Row-Based Fault-Tolerant Dynamic Operation Units for RRAM Computing-in-Memory Systems

**DOI:** 10.3390/mi17060708

**Published:** 2026-06-09

**Authors:** Liang-Ying Su, Zih-Yu Huang, Shih-Hsu Huang

**Affiliations:** Department of Electronic Engineering, Chung Yuan Christian University, Taoyuan 320314, Taiwan; g11202601@cycu.edu.tw (L.-Y.S.); 10826310@cycu.org.tw (Z.-Y.H.)

**Keywords:** crossbar array, integrated circuits, multiply-accumulate, reliability, redundant memories

## Abstract

Resistive random-access memory (RRAM) crossbar architectures are constrained by the limited number of word lines that can be activated simultaneously, thereby restricting the degree of parallelism achievable in computing-in-memory systems. To alleviate this architectural bottleneck, dynamic operation units have been proposed; however, existing implementations generally lack fault tolerance support, leading to significant yield degradation in the presence of defective memory cells. A recent study employed spare rows to provide fault tolerance, but such an approach is fundamentally limited by the number of available spare rows. In contrast, this paper proposes a generalized fault-tolerant scheme that is independent of the number of spare rows. To enable flexible row remapping, a novel word line activation generator are introduced. Experimental results demonstrate that the proposed architecture provides effective fault tolerance while incurring only minimal area and power overhead.

## 1. Introduction

As the scale of deep neural network (DNN) models continues to grow, a large volume of weights and feature data must be frequently transferred between computing units and external memory during inference, which gives rise to the memory wall problem [1,2,3,4,5,6]. This bottleneck, caused by the mismatch between computational speed and memory access bandwidth, has become a key factor that limits system performance and energy efficiency. To reduce the cost of data movement, computing-in-memory (CIM) architectures perform computations directly within memory arrays, thereby effectively improving data reuse and reducing the time and energy overhead associated with data transfer. Among various CIM technologies, resistive random access memory (RRAM) has emerged as one of the most promising CIM implementations because of its non-volatility, low power consumption, high density, and capability to perform in situ vector-matrix multiply-accumulate (MAC) operations in crossbar arrays.

**Figure 1:**
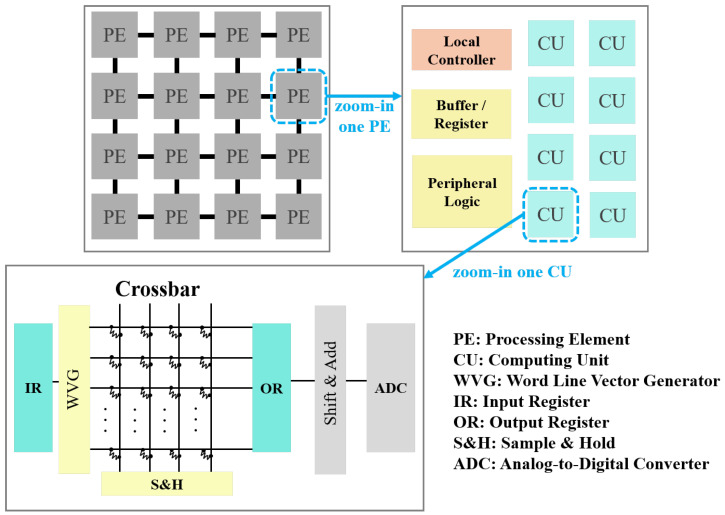
An illustration of a CU in an RRAM-based CIM system.

An RRAM-based CIM system typically consists of multiple processing engines (PEs), where each PE contains several computation units (CUs), and each CU is further composed of multiple crossbar arrays and their peripheral circuits, as shown in Figure 1. These peripheral components include, for example, the input register (IR), word line vector generator (WVG), sample-and-hold circuit (S&H), output register (OR), and analog-to-digital converter (ADC) [7]. Although such an architecture can efficiently execute large-scale matrix-vector multiplication, the simultaneous activation of a large number of word lines and bit lines to improve throughput is often constrained by the resolution and conversion speed of the ADC. A high-resolution ADC can improve computational accuracy, but it incurs higher area and power costs. In contrast, a low-resolution ADC is more favorable for energy saving and hardware implementation, but it can suffer from reduced accuracy and limited efficiency [8]. To achieve a balance among accuracy, throughput, and hardware cost, the concept of an operating unit (OU) was proposed in [9], in which a large crossbar array is divided into multiple smaller sub-computing units so that each OU activates only a fixed number of word lines and bit lines in a single cycle and performs dot-product operations in a segmented manner using low-resolution ADCs [9,10,11,12]. This approach can effectively reduce the accumulated current deviation and shorten the sensing latency.

However, the weights and activations in DNNs are typically highly sparse. If redundant computations associated with zero values can effectively be skipped, power consumption can be further reduced, and overall computational efficiency can be improved [9,10,13,14,15,16]. Based on this observation, the dynamic OU formation mechanism (DOF) was proposed in [9]. By exploiting the sparsity of input activations, DOF dynamically selects word lines with nonzero activations in each computation cycle to form a virtual OU, thereby skipping all-zero paths and reducing both execution cycles and energy consumption. In addition, this mechanism can be combined with sparse data representation, zero skip, or compressed data encoding techniques to further improve system performance [9,14,15,17].

As process variation increases and array sizes continue to scale up, non-ideal effects and device failures in RRAM crossbar arrays have become increasingly significant [18]. Common stuck-at-zero and stuck-at-one faults may prevent memory cells from correctly updating their resistance states, thereby causing weight storage errors and ultimately affecting the correctness of MAC operations. To address this issue, Huang et al. [19] proposed a fault-tolerant method that introduces spare rows into the dynamic OU architecture. By replacing faulty word lines with spare word lines, the availability and yield of RRAM-based CIM systems can be improved. Nevertheless, this method mainly considers the case where the demand for fault activation does not exceed the available redundancy resources, and it does not address the operating mechanism when the number of faulty word lines that need to be activated simultaneously in a given computation cycle exceeds the number of spare rows.

We observe that for the OU/DOF architecture, the sufficiency of spare rows is determined not by the total number of faulty word lines, but rather by the number of faulty word lines that must be activated simultaneously within a given cycle. To formally describe this issue, let *S* denote the number of spare rows allocated in each OU, and *F* denote the number of faulty word lines that need to be activated concurrently during a computation cycle. When F≤S, the method proposed in [19] can successfully perform fault mapping (to spare rows) and generate the corresponding activation vectors for all faulty word lines. However, when F>S, a single mapping process is no longer sufficient to cover all faulty activation requirements. In other words, whenever a cycle encounters the condition F>S, the method in [19] cannot operate correctly.

To address the above issue, this paper proposes an OU/DOF control architecture that dynamically performs mapping between faulty word lines and spare rows, allowing support for both fault scenarios where F≤S and F>S. When F≤S, the control logic can complete the mapping of the faulty word lines (to the spare rows) and the generation of the activation-vector in a single pass. When F>S, the proposed architecture employs a time-domain partitioning (multi-pass) strategy to divide the activation requirements (for faulty word lines) into multiple passes for sequential processing, ensuring that the number of spare rows used in each pass does not exceed *S*. Based on this mechanism, the original DOF scheme is modified to support segmented control. Under the constraint of the simultaneously activated word lines, valid word lines are dynamically selected to form virtual OUs, thereby maintaining a correct and stable computation flow.

Note that in addition to the design of the RRAM system, many studies have focused on RRAM materials, such as those reported in [20,21]. Furthermore, RRAM devices can serve as adaptive sensory front ends for neuromorphic edge intelligence, as demonstrated in [22].

The main contributions of this paper are summarized as follows. First, this paper proposes a fault-tolerant control mechanism that supports cases where the number of faulty rows exceeds the number of spare rows. As long as the spare rows themselves are fault-free, the proposed multi-pass remapping scheme can ensure the continued operation of the RRAM-based CIM system. Second, this paper further presents a hardware architecture to realize the proposed mechanism, including fault-aware vector remapping, prefix-sum correction, and a multi-pass control module. Finally, the experimental results show that the proposed method incurs only very small areas and power overhead, demonstrating its strong feasibility for hardware implementation and practical application potential.

## 2. Background

### 2.1. RRAM-Based CIM

RRAM has been widely adopted in CIM architectures to support data-intensive operations such as vector-matrix multiplication due to its non-volatility, high integration density, and low power consumption [23,24,25,26,27,28]. Unlike the conventional von Neumann architecture, which requires frequent data transfers between the processor and external memory, RRAM-based CIM can directly perform MAC operations within crossbar arrays, effectively reducing the latency and energy consumption caused by data movement.

In RRAM-based CIM, weights are typically stored in crossbar arrays in the form of conductance values. When input voltages are applied to the word lines, the currents generated by the corresponding memory cells accumulate along the bit lines, thereby completing the analog dot-product operations [2,8,24]. The overall system is usually integrated with peripheral circuits such as digital-to-analog converters (DACs), analog-to-digital converters (ADCs), and shift-and-add units to support multi-bit input and output accumulation. Since the resolution, speed, area, and power consumption of the ADC significantly affect the overall design, how to reduce the cost of the ADC while maintaining computational correctness has become an important issue in the design of RRAM-based CIM architectures.

### 2.2. Operation Unit

To reduce the dynamic range of the accumulated bit line current, the concept of an operation unit (OU) has been proposed in the literature [9] as a segmented computing mechanism in the time domain in RRAM-based CIM. For the *i*-th bit line, the output current can be expressed as

(1) $$I_{BL,i}\;=\;\sum_{j\;\in \;A}G_{ij}\;V_{j},$$

where $G_{ij}$ denotes the conductance stored in the $\left(i,j\right)$-th cell, $V_{j}$ denotes the input voltage applied to the *j*-th word line, and A represents the set of word lines activated in that cycle. As the number of simultaneously activated word lines increases, the dynamic range of the accumulated bit line current also increases, requiring the ADC to provide higher resolution to avoid saturation or quantization errors, which in turn increases the area and power cost. To address this issue, the OU activates only a fixed number of word lines and bit lines in each computation cycle through control logic such that the system performs local dot-product operations under a constrained computation scale. In this way, the range of the bit line current can be effectively reduced and the ADC burden can be alleviated.

**Figure 2:**
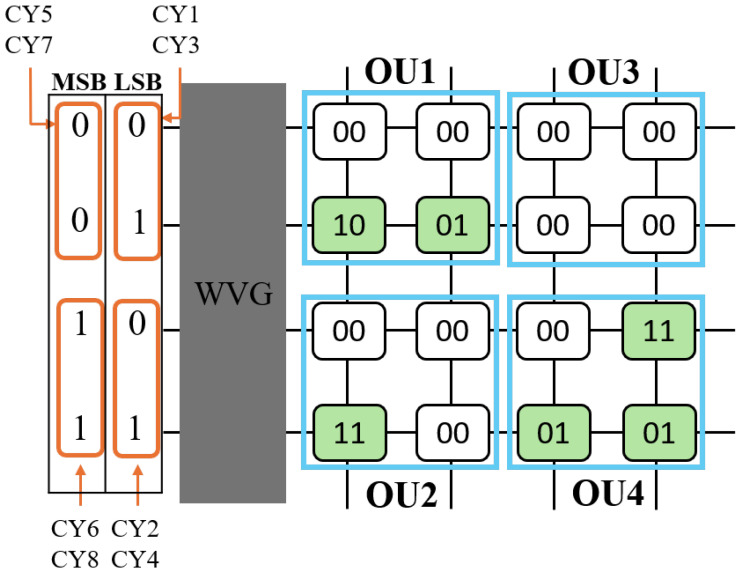
An illustration for the operation of the OUs.

As shown in Figure 2, the OU can be regarded as a logical sub-block configuration on the crossbar array. The system sequentially activates the corresponding OUs in different cycles to complete the overall computation. To support multi-bit operations, bit-slicing and shift-and-add schemes can be further employed to combine the partial results generated in each cycle into the final output.

Figure 2 illustrates the operation of the OUs. Since the input activation is 2 bits, it is divided into the most significant bit (MSB) and the least significant bit (LSB). In this example, we assume that each OU has a size of 2 × 2. Therefore, the crossbar array consists of four OUs: OU1, OU2, OU3, and OU4. During cycles 1 to 4 (i.e., CY1–CY4), the system sequentially feeds the LSB data into OU1 through OU4 for computation. During cycles 5 to 8 (i.e., CY5–CY8), the system sequentially feeds the MSB data into OU1 through OU4 for calculation.

**Figure 3:**
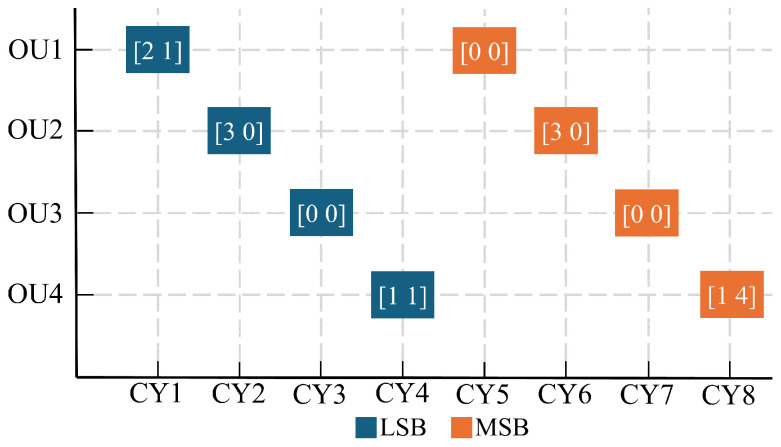
The computation results in each cycle.

Figure 3 illustrates the computation results in each cycle. For example, in cycle 1, the computation results of the two bit lines in OU1 are 2 and 1; in cycle 2, the computation results of the two bit lines in OU2 are 3 and 0; and so on. Note that shift-and-add schemes can be further employed to combine the partial results generated in each cycle into the final output. Through this mechanism, the OU provides a means of shedding computational parallelism, ADC resolution requirements, and hardware cost, and has become one of the commonly adopted design techniques in RRAM-based CIM architectures.

### 2.3. Dynamic OU Formation

**Figure 4:**
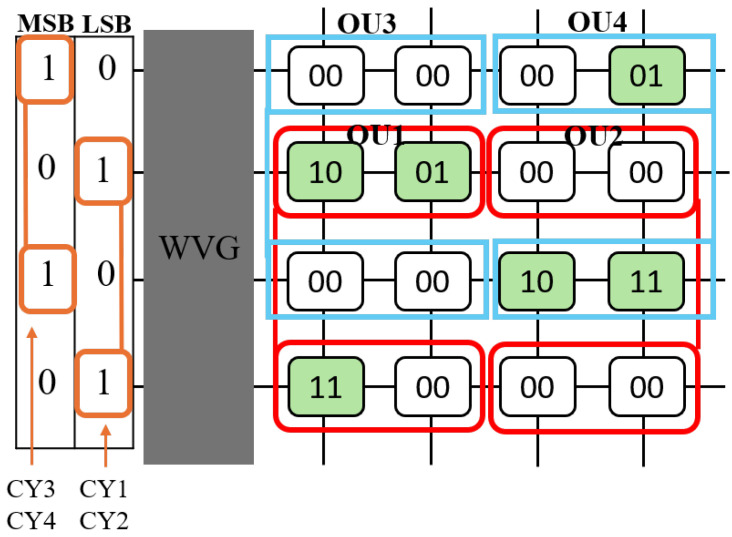
OU configuration under the DOF architecture.

Since weights and activations in deep neural networks are often highly sparse, if a fixed OU schedule is still adopted, the system may continue to activate the corresponding computations in a predetermined manner even when some word lines are associated with zero-valued inputs, thereby resulting in redundant cycles and additional energy consumption. To address this issue, Dynamic OU Formation (DOF) was proposed in [9], as shown in Figure 4, allowing the system to dynamically select effective word lines according to the sparsity of input and form a virtual OU.

In the DOF architecture, the number of word lines that can be activated simultaneously in each cycle is limited. Here, $S_{WL}$ denotes this upper bound. A fixed-dimension 1D mask vector and a prefix-sum circuit are used to generate the activation vector to construct a virtual OU. Specifically, the system first generates a mask vector according to the input vector to identify the word lines corresponding to nonzero inputs. Let $x_{j}$ denote the input of the *j*-th word line. Here, the mask vector can be expressed as $m_{j}=x_{j}$, where $m_{j}$ denotes the mask bit of the *j*-th word line. If the input of the *j*-th word line is zero, then $m_{j}=0$; if the input of the *j*-th word line is nonzero, then $m_{j}=1$.

Next, the system performs a prefix-sum operation on the mask vector to obtain the following:

(2) $$p_{j}=\sum_{k=0}^{j}m_{k},$$

where $p_{j}$ denotes the accumulated number of effective word lines up to and including the *j*-th word line. According to the maximum number of simultaneously activated word lines allowed by the OU, the system defines a range bound [L,H) and selects the word lines that meet the following:

(3) $$L\leq p_{j}<H,$$

to form the virtual OU of the current cycle, where $H=L+S_{WL}$. This method transforms the original OU structure based on fixed spatial partitioning into a dynamic time-domain mapping problem, such that effective word lines can be executed in batches over multiple cycles.

We use the example in Section 2.2 for illustration. As shown in Figure 4, for the computations associated with the LSB of the input activation, only the second and fourth word lines correspond to nonzero inputs. Therefore, the second and fourth word lines can form two dynamic OUs, i.e., OU1 and OU2. In other words, the system requires only two cycles (i.e., CY1 and CY2) to complete the computations for the LSB of the input activation.

For the computations associated with the MSB of the input activation, only the first and third word lines correspond to nonzero inputs. Therefore, the first and third word lines can form two dynamic OUs, i.e., OU3 and OU4. In other words, the system requires only two cycles (i.e., CY3 and CY4) to complete the computations for the MSB of the input activation.

**Figure 5:**
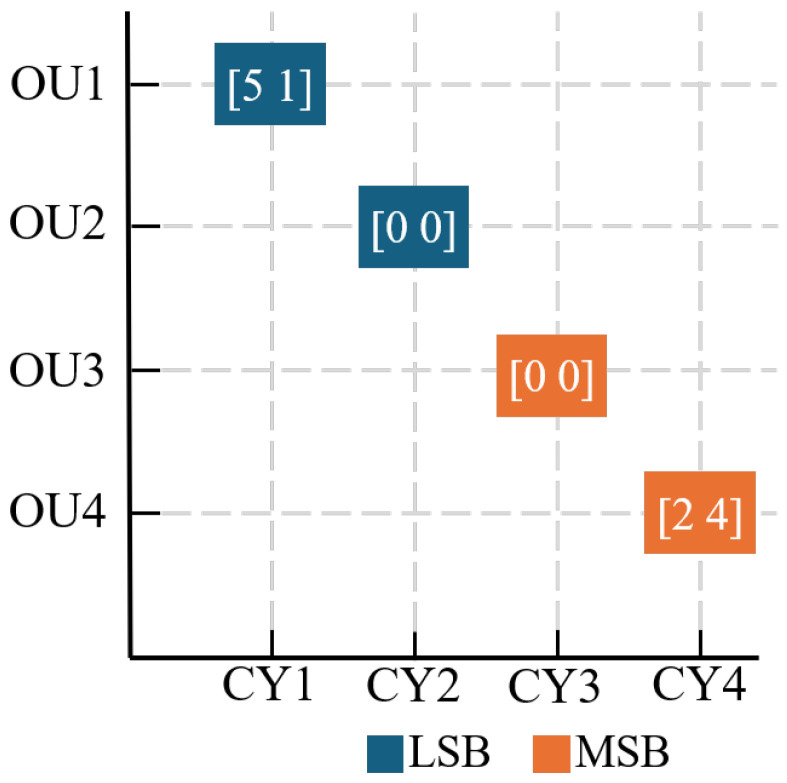
Dynamic activation timing diagram of DOF.

Figure 5 displays the computation results in each cycle. In this example, the complete 2-bit dot-product operation can be completed in only four cycles. Compared with the fixed OU architecture (i.e., Figure 2), the DOF architecture in this example reduces the number of computation cycles from 8 to 4.

From a system-level perspective, DOF preserves the advantages of the OU architecture in controlling the dynamic range of current and reducing ADC cost while significantly reducing redundant cycles through a sparsity-aware scheduling strategy. Under high-sparsity conditions, this mechanism can effectively improve both computational efficiency and energy efficiency.

### 2.4. Word Line Vector Generator

To support the dynamic word line selection flow of the DOF, Yang et al. [9] propose a Word Line Vector Generator (WVG) architecture as the core control module to generate the word line activation vector for the DOF architecture. Its function is to detect zero-values, calculate the prefix-sum, and match the range defined in Equations (2) and (3) under constraint $S_{WL}$, and output the corresponding control signals of the word line.

**Figure 6:**
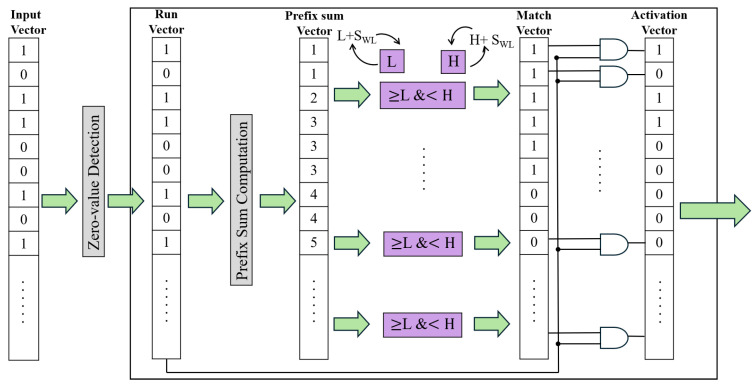
The original WVG data path for DOF [9].

As shown in Figure 6, the WVG data path for the DOF architecture [9] can be divided into four stages:

1. Zero-value Detection: If the input value is multi-bit, zero-value detection is performed to determine whether the input value is zero or nonzero. This detection can be implemented using multi-bit OR-reduction logic. The detection result is then stored in the Run Vector. In Figure 6, we assume that the input value in the input vector is a 1-bit value.
2. Prefix-Sum Computation: A prefix-sum operation is performed on the mask vector to generate $p_{j}$. This step can be implemented using cascaded adders, a scan network [29], or a parallel-prefix adder architecture, depending on the tradeoff between the target frequency and the area requirements.
3. Range Matching: The controller provides the bound [L,H). A comparator array determines for each word line whether its $p_{j}$ falls within this range, thus achieving the decision defined in (2).
4. Activation Vector Generation: After logic integration, the matching results are output as the word line activation vector $a_{j}$, which directly drives the word line selection circuitry of the crossbar array to form the virtual OU in the current cycle.

The WVG data path as shown in Figure 6 converts the algorithm-level word line selection flow of the DOF into a hardware-realizable control mechanism, allowing dynamic OU formation under a fixed constraint $S_{WL}$.

## 3. Motivation

Although RRAM-based CIM has been widely regarded as a promising technology for accelerating deep neural network inference due to its high density, low power consumption, and in-memory computing capability, its insufficient process maturity and device non-idealities still pose significant challenges to practical deployment. At the device level, common fault types include stuck-at faults, such as stuck-at-zero and stuck-at-one faults, which prevent the resistance states of memory cells from being correctly updated and thereby lead to weight errors.

In addition, phenomena such as resistance drift, write variation, and IR-drop within the array can further amplify the analog MAC errors, thus deteriorating overall system reliability [30,31,32,33]. In large-scale array and high-density deployment scenarios, how to maintain computational correctness and system availability under limited hardware overhead has become an important issue in the design of RRAM-based CIM systems.

To address the above challenges, various cross-layer approaches have been proposed, such as avoiding faulty cells through fault-aware weight mapping, improving yield through hardware redundancy, or balancing performance and energy efficiency through dynamic precision control [8,11,18,19,34,35]. However, for architectures employing OU and DOF, previous work [9] has mainly focused on exploiting sparsity and improving computational efficiency, while a comprehensive investigation into maintaining DOF scheduling consistency and activation correctness under fault conditions remains lacking. To our knowledge, ref. [19] is the only study that specifically addresses fault-tolerant design for the DOF architecture.

In [19], spare rows are introduced into the dynamic OU architecture such that faulty word lines can be replaced by spare word lines, thus improving the availability and yield of RRAM-based CIM systems. However, when the system allocates *S* spare rows for fault tolerance, this method implicitly assumes that the number of activated faulty rows to be replaced in a given computation cannot exceed the redundancy capacity, i.e., F≤S, where *F* denotes the number of faulty rows that are activated simultaneously during the current computation cycle. Under this condition, the faulty rows can be remapped to spare rows using the fault tolerance mechanism proposed in [19]. However, in practical computing scenarios, the number of activated faulty rows within a computation cycle may exceed the number of spare rows; that is, the condition F>S may occur. However, Huang et al. [19] do not address this issue. There is a need to develop a comprehensive fault-tolerant mechanism that can handle all possible scenarios; that is, it should be able to deal with both F≤S and F>S conditions simultaneously.

Motivated by this observation, in this paper, we propose a comprehensive fault-tolerant mechanism that converts excessive fault demand in the activation domain into a time-domain segmented computation problem. When F≤S, the system maintains the single-pass execution. When F>S, a multi-pass mechanism is employed to process the faulty activated rows in batches, and the results of all passes are accumulated in the digital domain to ensure that the final computation result remains equivalent to that of the original input.

Compared with previous work [19], which only addresses the case where the faulty activation demand does not exceed the capacity of the spare row, this work further supports scenario F>S while preserving system correctness and scalability without modifying the original DOF core control flow.

Our work aims to establish a comprehensive fault-tolerant mechanism for the DOF architecture, enabling RRAM-based CIM systems to maintain functional correctness and high reliability under a limited number of spare rows. In particular, the key feature of our approach is that the system can still provide a correct computation even when the condition F>S occurs.

## 4. Proposed Approach

To improve the resilience and stability of RRAM-based CIM architectures in the presence of defective word lines, this work proposes a fault-tolerant word line activation vector architecture. The proposed method performs real-time correction of the input vector through spare-row allocation and vector-correction logic, and then combines the corrected sparse mask with the prefix-sum results to generate the corresponding word line activation vector.

When the number of activated faulty word lines (for the current calculation), indicated by *F*, does not exceed the number of spare rows *S* (i.e., F≤S), the proposed mechanism can complete the correction and activation of the input before the beginning of a single calculation pass. As a result, the original MAC execution flow does not need to be interrupted and neither the $S_{WL}$ constraint nor the dynamic OU formation mechanism of the DOF architecture is altered. In this way, the system can still maintain computational correctness and high throughput efficiency even in the presence of hardware faults.

When F>S, the system instead adopts the subsequently proposed multi-pass mechanism to perform segmented computation, thereby ensuring functional correctness while exhibiting predictable delay degradation behavior in the time domain.

### 4.1. The Use of Spare Rows

This work reserves *S* spare rows in each OU crossbar array as replacement resources for faulty word lines. When the detection circuit finds during a write or read operation that the conductance value of a main word line persistently deviates from the expected range, the system identifies that word line as having a permanent or intermittent fault and marks it in the fault indicator vector. To describe the activation demand, the following definitions are used.

- Fault indicator vector f[i]: if the *i*-th word line is faulty, then f[i] = 1;
- Input vector x[i]: indicates whether that *i*-th word line needs to be activated in the calculation process.

For the *i*-th word line, if f[i]=1, we refer to this word line as a faulty word line. In fact, not all faulty word lines require the use of spare row resources. For the *i*-th word line, if f[i]=1 and x[i]=1, we refer to this word line as an activated faulty word line. In other words, only activated faulty word lines require spare row resources. We define *F* as the number of activated faulty word lines in an OU calculation. Therefore, the number of spare rows required for an OU calculation is F.

We need to prevent faulty word lines from participating in OU computations to prevent incorrect data (i.e., erroneous weight values) from being involved in the calculation. To maintain computational correctness, the control logic must relocate the data originally assigned to faulty word lines to spare rows, and then allow the spare rows to participate in the computation.

To indicate the word lines that should actually participate in the computation, we define a Mask Vector as follows. For the *i*-th word line, if f[i]=0 and x[i]=1, then we have Mask[i]=1; otherwise, we have Mask[i]=0.

**Figure 7:**
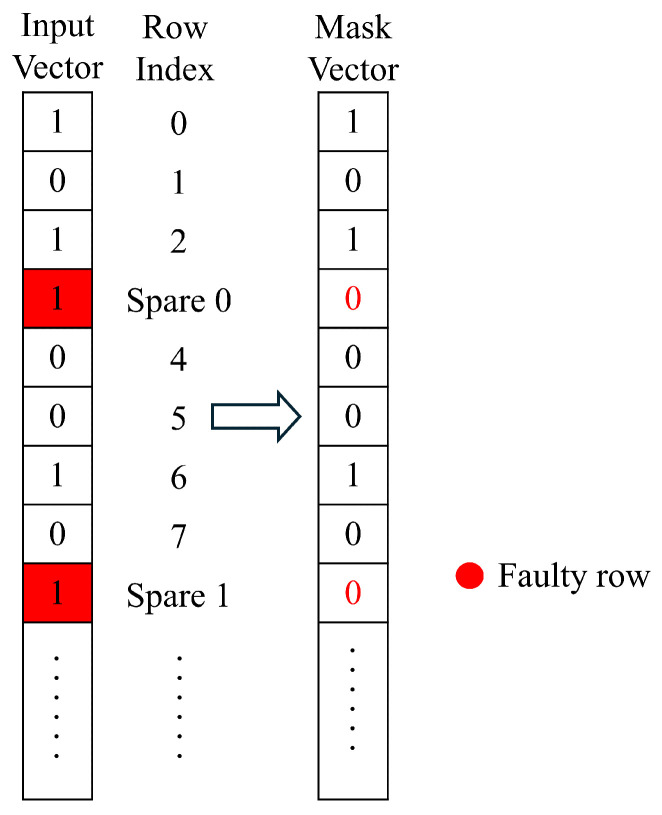
Input vector correction flow considering spare rows.

As shown in Figure 7, this example contains two faulty word lines. The data originally assigned to the faulty word lines are relocated to spare rows. Since the third and eighth word lines are faulty, we have Mask[3]=0 and Mask[8]=0.

If the number of faulty word lines exceeds the number of spare rows, a multi-pass mechanism must be employed. We define a Run Vector to denote the word lines participating in the computation in each pass. For the *i*-th word line, if f[i]=0 (i.e., the *i*th word line is a normal word line), then we have Run[i]=x[i] in the first pass and Run[i]=0 in all subsequent passes.

Furthermore, during each pass, the control logic schedules *S* activated faulty word lines (i.e., word lines that satisfy f[i]=1 and x[i]=1) to participate in the computation, where *S* is the number of spare rows. For an activated faulty word line (assuming that it is the *i*-th word line), we have Run[i]=1 only in the pass where it is scheduled to participate in the computation; in all other passes we have Run[i]=0.

In other words, we use the Run Vector as the input for each pass. Obviously, if the number of activated faulty word lines does not exceed the number of spare rows (i.e., F≤S), the computation can be completed in a single pass. Therefore, in this case, the Run Vector is identical to the Input Vector. However, if the number of activated faulty word lines exceeds the number of spare rows (i.e., F>S), the control logic must update the Run Vector in each pass to complete the computation. The total number of passes is 1+⌈(F−S)/S⌉.

### 4.2. Prefix-Sum Correction

The prefix-sum operation is the core logic in the DOF architecture to determine which word lines should be activated in each clock cycle. Its purpose is to transform the input vector into a cumulative activation index, thereby supporting subsequent word line activation and dynamic OU formation. After introducing spare rows, this work extends the prefix-sum computation flow in a fault-aware manner to ensure that the statistical ordering remains consistent with the fault-free case even in the presence of faults.

**Figure 8:**
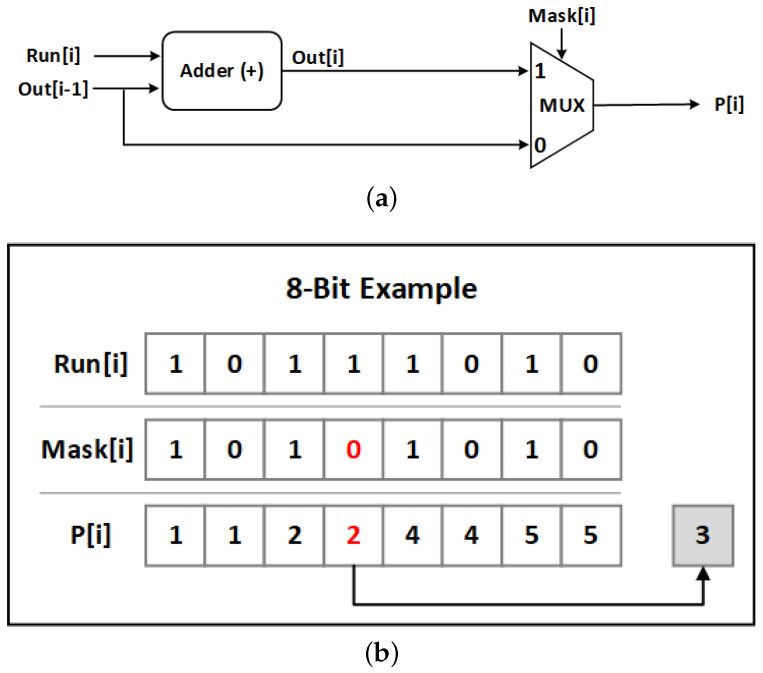
Fault-aware prefix-sum correction mechanism. (**a**) The data path for a single word line. (**b**) An 8-bit example.

Figure 8a shows the prefix-sum update data path for a single word line. In our design, the Run Vector serves as the input for each pass. For the *i*-th word line, Run[i] is added to Out[i−1] to generate a candidate cumulative result Out[i]. The output P[i] is then selected through a multiplexer controlled by Mask[i]. If Mask[i]=1, the corresponding word line is valid and allowed to participate in the counting process, and the output is updated to the accumulated result Out[i]. If Mask[i]=0, the previous accumulated value is directly bypassed, such that P[i]=Out[i−1], thereby avoiding incorrect accumulation at unavailable positions, such as faulty word lines. This design allows faulty positions to be treated as “holes” that are excluded from the counting process, while maintaining the consistency and predictability of the prefix-sum results.

Figure 8b provides an 8-bit example to illustrate the prefix-sum correction under fault conditions and spare-row remapping. The Run Vector is given as Input[i]=[1,0,1,1,1,0,1,0]. Among these entries, the fourth position originally has Run[i]=1, indicating that the corresponding word line is required to be activated. However, since the word line is faulty, Mask[i] is set to 0 (the red 0 in the figure). When Mask[i]=0, the prefix-sum computation bypasses the accumulation update at that position, so the output P[i] remains equal to the previous value (the red P[i]=2 in the figure), thus preventing the unavailable position from being counted and causing incorrect indexing.

At the same time, this activation demand is not discarded. Instead, it is remapped to a spare row and the corresponding cumulative index is restored at the spare position (marked as 3 in the figure). This indicates that the logical activation order is immediately after the cumulative value 2, becoming the next valid activation index. Since the missing accumulation has been compensated for at the spare location, the prefix-sum values of the subsequent available positions can continue to follow the correct statistical sequence (e.g., the later outputs become 4, 5, and so on). This behavior is equivalent to not accumulating in the faulty position, while compensating for the missing count in the spare position, thus ensuring that the statistical ordering of the prefix sum remains consistent with the fault-free case even in the presence of faults and mapping. Consequently, the correctness and stability of the DOF architecture can be preserved.

### 4.3. Word Line Activation

After the prefix-sum correction is completed, the system dynamically generates the Word Line Activation Vector for each computation cycle by combining the corrected prefix-sum results with the mask vector. The detailed decision flow is described below.

1. Bounds Checking
  For the prefix-sum result $P_{j}$ of the *j*-th word line, the system determines the allowable activation range in the current cycle according to the current cycle index c and the number of word lines that can be activated simultaneously in an OU, denoted by $S_{WL}$. Taking a 2×2 OU as an example, i.e., 2 word lines × 2 bit lines, and setting $S_{\mathrm{WL}}=2$, the boundary conditions are defined as follows.
  - Lower Bound Check: determines whether the cumulative index reaches the starting threshold of the current cycle:
    $$L_{j}=\left\{\begin{array}{cc} 1, & \;\mathrm{if}\;P_{j}\geq 1+\left(c-1\right)\times S_{WL}\\ 0, & \;\mathrm{otherwise}\; \end{array}\right\}$$
  - Upper Bound Check: determines whether the cumulative index falls within the ending threshold of the current cycle:
    $$H_{j}=\left\{\begin{array}{cc} 1, & \;\mathrm{if}\;P_{j}<1+c\times S_{WL}\\ 0, & \;\mathrm{otherwise}\; \end{array}\right\}$$
2. Activation Decision
  The results of the lower-bound and upper-bound checks are then logically ANDed to produce the Match Vector:
  $$Match\left[j\right]=L_{j}\wedge H_{j}$$
  We can use both the Mask Vector and the Match vector to determine whether the corresponding word line should finally be activated:
  $$Activation\left[j\right]=\left\{\begin{array}{cc} Mask\left[j\right]\wedge Match\left[j\right], & \;\mathrm{if}\;j-\mathrm{th}\;\mathrm{word}\;\mathrm{line}\;\mathrm{is}\;a\;\mathrm{normal}\;\mathrm{word}\;\mathrm{line}\\ Match\left[j\right], & \;\mathrm{if}\;j-\mathrm{th}\;\mathrm{word}\;\mathrm{line}\;\mathrm{is}\;a\;\mathrm{spare}\;\mathrm{word}\;\mathrm{line}\; \end{array}\right\}$$
3. Output Control Signal
  If the activation condition is satisfied, i.e., Activation[j]=1, the corresponding word line is selected and activated. The control logic then outputs the corresponding control signal to drive either the main word line or the spare word line to perform the MAC operation in the current cycle. Through this activation decision mechanism, the system can automatically avoid the word lines associated with defective cells while preserving the dynamic virtual-OU formation behavior. Since the prefix-sum results have already been corrected in a fault-aware manner in the earlier stage, the activation ordering remains consistent even in the presence of faults, and thus the overall MAC computation result is not affected. When F≤S, all faulty activation demands can be remapped and handled in a single pass, so the system performance remains unchanged. When F>S, the computation is partitioned in the time domain through the multi-pass overflow mechanism, while each pass still follows the same activation decision logic.

### 4.4. Overall Architecture

**Figure 9:**
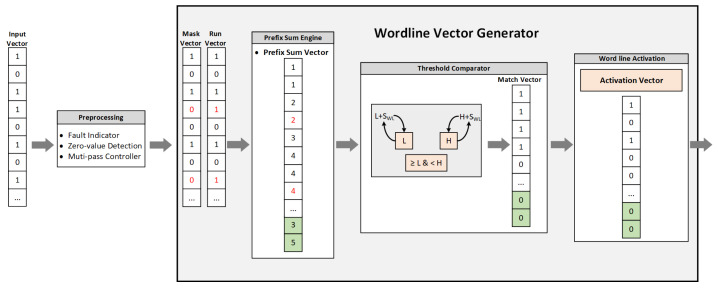
Proposed fault-tolerant WVG data path for DOF.

As shown in Figure 9, the integrated architecture proposed in this work can be regarded as an improved version of the original WVG architecture proposed in [9]. While preserving flow based on existing DOF and constraint $S_{WL}$, the proposed architecture incorporates fault-based front-end control and vector correction/mapping mechanisms into the vector generation chain, allowing the system to maintain consistent word line selection semantics and predictable performance behavior under different fault loads. After the Input Vector enters the front end, the overall data flow proceeds through spare-constrained candidate generation (i.e., Run Vector generation), prefix-sum-based calculation, and threshold-window comparison to produce the Match Vector, and finally outputs the Word Line Activation Vector, which can directly drive the word lines.

The processing flow begins with the Preprocessing unit, which receives faulty word line information provided by an external test or diagnosis mechanism and uses it as the basis for subsequent fault-aware mask generation and word line selection control. In this work, the Fault Indicator denotes the physical fault-location information of the main word lines established by the external detection or diagnosis procedure. The Fault Indicator serves as the cycle/pass-level control vector used in the WVG vector-generation flow. In this work, we assume that the required fault information is available from external mechanisms and focus on how to utilize this information for fault-aware masking, remapping, and activation vector generation. Using the Fault Indicator, the downstream vector generation flow can automatically avoid unavailable resources, thereby preventing faulty word lines from being included in the valid activation set or causing incorrect accumulation.

Next, the Multi-pass Controller determines whether the computation should proceed in single-pass or multi-pass mode. When the activation demand can be satisfied within a single pass, the system directly completes prefix sum calculation, filtering, and activation vector generation in single pass mode. When segmentation is required due to redundancy resource limitations, the Multi-pass Controller generates a Pass Index, allowing the input vector to be processed sequentially across multiple passes until the required coverage is completed. This multi-pass mechanism is mainly used to coordinate the candidate-subset activation (i.e., Run Vector), without modifying the computational structure of the downstream data path. Therefore, regardless of whether the system operates in single-pass or multi-pass mode, the external vector width and interface format remain unchanged; the only difference lies in the candidate subset activated in each pass.

Under the constraints imposed by the Fault Indicator and, in the multi-pass case, the Pass Index, the vector-correction and remapping mechanism performs fault-aware processing on the candidate-subset activation (i.e., Run Vector). The positions corresponding to faulty word lines are effectively suppressed in the subsequent prefix sum counting process, and when it is necessary to preserve the activation semantics, the corresponding activation demand is redirected to the spare rows, thereby forming an effective candidate set that satisfies the fault tolerance constraints. This design ensures that the input content used for subsequent ranking and selection already complies with the fault-tolerant requirements. Moreover, under multi-pass operation, the equivalent activation coverage can be progressively completed in the time domain by different passes.

In each pass, the Run vector is sent to the Prefix Sum Engine, where the accumulation of prefix sums is performed to generate the activation ranking. This step maps the candidate word lines into comparable ranking information while preserving the original relative ordering of the inputs, thereby providing a common basis for subsequent word line selection decisions. Since the computational structure of the Prefix Sum Engine remains unchanged under different fault conditions as well as in both single-pass and multi-pass modes, and the only difference is reflected in the effective input after fault-aware processing, the DOF dynamic word line selection flow can maintain consistent control semantics even under fault scenarios.

The ranking results are then compared with the L/H threshold window by the Threshold Comparator, which generates the Match Vector to indicate which candidate word lines fall within the valid activation interval. Finally, the Word Line Activation Logic combines the Match Vector and the Mask Vector to produce the final Word Line Activation vector, which is used to drive either the main word lines or the spare word lines to perform the MAC operation. Overall, while preserving the high-efficiency sparse-control capability of the original DOF architecture, the proposed architecture enhances the adaptability of the system to faulty devices through fault-aware vector correction/remapping and simplified segmented scheduling control, thereby enabling reliable and scalable operation even in high-fault-rate environments.

### 4.5. Fault-Aware Multi-Pass Word Line Activation Algorithm

When the number of activated faulty rows F exceeds the number of spare rows S, the system cannot accommodate all the demands of activated faulty rows in a single pass. To address this issue, we present a multi-pass mechanism. The core idea is to distribute the demand for activated faulty rows in different passes. Each pass subsequently generates its corresponding Run Vector, Prefix Sum calculation, Match Vector, and Activation Vector, thereby preserving the correct activation behavior in the DOF architecture under fault conditions. To more precisely describe the proposed multi-pass control mechanism, Algorithm 1 details the control flow and operational procedures of the proposed scheme.
**Algorithm 1** Fault-Aware Multi-Pass Word Line Activation**Input:** Input Vector x[i], Fault Indicator f[i], Number of Spare Rows *S***Output:** Activation Vector for Each Pass  1:Generate Mask Vector from Input Vector and Fault Indicator;  2:Calculate F (i.e., number of activated faulty rows) from Input Vector and Fault Indicator;  3:Determine the number of passes:  4:     if F≤S then totalpass=1  5:     else totalpass=1+⌈(F−S)/S⌉;  6:For index = 1 to totalpass do {  7:     Generate Run Vector R[i] for this pass;  8:     Remap the data of selected faulty rows to spare rows;  9:     Compute Prefix Sum Vector according to Run Vector;10:     Let *U* be the largest value among Prefix Sum Vector;11:     Let L=1 and $H=L+S_{\mathrm{WL}}$;12:     While (L≤U) do {13:        Generate Match Vector according to Prefix Sum Vector and window [L,H):14:        Generate Activation Vector according to Match Vector and Run Vector;15:        Let $L=L+S_{\mathrm{WL}}$ and $H=H+S_{\mathrm{WL}}$; } }

In Table 1, we use an example to demonstrate the process of Algorithm 1. In this example, we assume that the number of spare rows is 2 and that the CIM array contains 16 word lines. From the fault indicator and the input vector, this example contains four activated faulty word lines. Since the number of activated faulty word lines exceeds the number of spare rows, the multi-pass control scheme must be employed. As shown in Table 1, in this example, a total of two passes (i.e., 1+⌈(4−2)/2) are required.

In Pass 1, the Run Vector is generated to handle all activated normal word lines and two activated faulty word lines, due to the limitation of having only two spare rows. The prefix-sum vector is then calculated, where the largest value in the prefix-sum vector is 7. In this example, we assume that the maximum number of simultaneously activated word lines in the DOF architecture is constrained to 2. Therefore, in this pass, the generation of the Match Vector and the Activation Vector is divided into four stages: (L = 1, H = 3), (L = 3, H = 5), (L = 5, H = 7), and (L = 7, H = 9). The details of the Match Vector and the Activation Vector for each stage are presented in Table 1.

In Pass 2, the Run Vector is generated to handle the remaining activated faulty word line. The prefix-sum vector is then calculated, where the largest value in the prefix-sum vector is 2. Therefore, in this pass, the generation of the Match Vector and the Activation Vector consists of only one stage: (L = 1, H = 3). The details of the Match Vector and the Activation Vector are displayed in Table 1.

Note that although each pass generates its corresponding Activation Vector at different time points, the overall effect, after digital-domain accumulation, remains equivalent to the activation demand required by the computation. In this way, functional correctness and consistency of the control semantics can still be maintained even when the number of spare rows is insufficient.

The efficiency of the proposed method depends on the relationship between the number of activated faulty word lines (i.e., F) and the number of available spare rows (i.e., S) in each dynamically formed OU. If every dynamic OU satisfies F≤S, the MAC operation can be completed in a single pass without invoking the multi-pass mechanism. In this case, the proposed method achieves the same efficiency as the previous fault-tolerant approach [19].

When a dynamic OU contains more activated faulty word lines than available spare rows, i.e., F>S, the proposed method activates the multi-pass mechanism to preserve computational correctness. Although this introduces additional passes, the previous fault-tolerant approach [19] cannot correctly complete the computation under this condition. Therefore, the proposed method is most efficient when faults are sparse or distributed such that most dynamic OUs satisfy F≤S, while it provides a correctness-guaranteed fallback when F>S.

In terms of latency, the proposed multi-pass mechanism introduces a time-domain trade-off when F>S. When F≤S, the computation can be completed in a single pass. When F>S, the activated faulty rows are partitioned into multiple passes so that each pass satisfies the available spare-row constraint. Therefore, the total latency can be approximated as

(4) $$L_{\mathrm{total}}=N_{\mathrm{pass}}\times L_{\mathrm{pass}},$$

where $N_{\mathrm{pass}}$ is the number of required passes and $L_{\mathrm{pass}}$ is the latency of one pass. According to the proposed control flow, $N_{\mathrm{pass}}=\left\lceil F/S\right\rceil$ when F>S.

It should be noted that $N_{\mathrm{pass}}$ is determined by the number of activated faulty rows rather than by the physical fault density alone. Since only activated faulty rows need to be remapped, input sparsity can effectively reduce the number of faulty rows involved in a given computation. As a result, even under a high physical fault density, the number of additional passes may remain small when the input activations are sparse. In contrast, the worst-case latency occurs when dense input activations coincide with a large number of faulty rows within the same computation window, thereby requiring multiple additional passes.

## 5. Experimental Results

To validate the proposed fault-tolerant WVG architecture, we compare the following three methods:

1. The original WVG architecture proposed in [9], which does not support fault tolerance;
2. The fault-tolerant WVG architecture proposed in [19] supports fault scenarios under the assumption that F≤S;
3. The proposed WVG architecture (i.e., Ours), which provides a comprehensive fault-tolerant mechanism.

The evaluation covers various CIM array sizes, including configurations with 16, 32, 64, 128, 256, 512, and 1024 normal word lines. For each CIM array size, different numbers of spare rows are considered, including configurations with 1, 2, 3, and 4 spare rows. All WVG architectures are described in Verilog RTL and synthesized using Synopsys Design Compiler based on TSMC 40 nm process technology. Power consumption is evaluated using Synopsys PrimeTime.

We verify the correctness of the proposed WVG architecture through simulation. The test patterns are generated by randomly creating combinations of Fault Indicators and Input Vectors. The generated Fault Indicators cover different fault densities, while the generated Input Vectors cover different sparsity densities. Consequently, the combinations of Fault Indicators and Input Vectors include both F≤S (single pass) and F>S (multiple passes) scenarios. The simulation results demonstrate that the proposed WVG architecture can produce correct MAC computation results in both the F≤S (single pass) and F>S (multiple passes) cases. Therefore, the proposed WVG design indeed provides a comprehensive fault-tolerant capability.

Note that the randomly generated fault indicators are used for functional verification only and are not intended to represent any specific RRAM defect distribution. Since the proposed row-remapping mechanism operates solely according to the detected fault locations, its correctness is independent of whether the faults are randomly distributed, clustered, or spatially correlated. Therefore, uniformly random fault patterns are sufficient for verifying the correctness of the proposed control logic.

**Figure 10:**
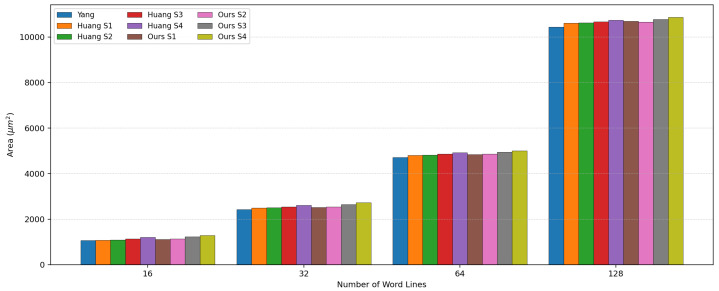
The circuit areas of different methods (for the number of word lines from 16 to 128).

**Figure 11:**
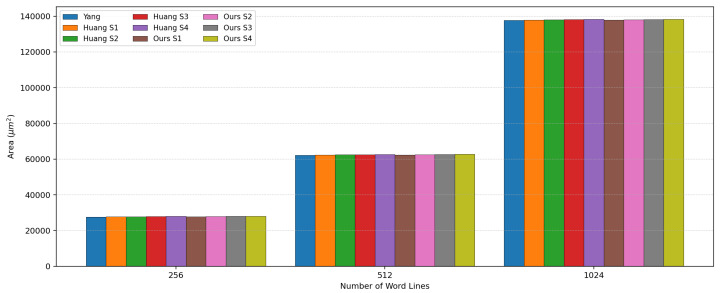
The circuit areas of different methods (for the number of word lines from 256 to 1024).

**Figure 12:**
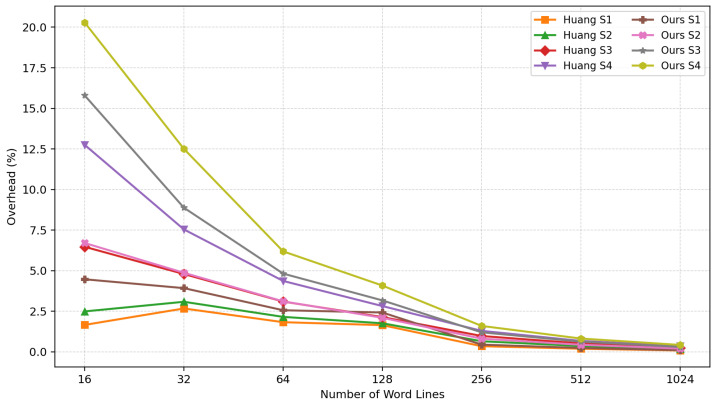
The percentage of additional area overhead introduced by the fault-tolerant mechanisms.

For the area analysis, Figure 10 and Figure 11 present the area comparison results of the three methods, including Yang et al. [9], Huang et al. [19], and ours, under different CIM array sizes. In these figures, we use S1, S2, S3 and S4 to represent the cases where the numbers of spare rows are 1, 2, 3 and 4, respectively. The results show that, compared to the original WVG architecture (i.e., Yang et al. [9]), both the previous fault-tolerant method (i.e., Huang et al. [19]) and the proposed fault-tolerant method (i.e., ours) introduce an additional area cost. Among them, the proposed method generally occupies a slightly larger area than that of Huang et al. [19] because it must additionally support the multi-pass control mechanism. We also observe that, as the number of word lines increases, the area of the original DOF architecture grows almost linearly, whereas the additional area introduced by fault-tolerant mechanisms (including Huang et al. [19] and ours) increases only slightly. Therefore, as the number of word lines increases, the area overhead of the fault-tolerant mechanisms is rapidly amortized. Figure 12 illustrates the percentage of additional area overhead introduced by fault-tolerant mechanisms for different numbers of word lines.

In Figure 12, the overhead is higher for smaller word-line sizes, mainly due to a normalization effect. The proposed method requires additional control logic for fault-aware selection, remapping, and multi-pass control to support fault tolerance. The area of these control modules does not scale proportionally with the number of word lines. In contrast, the area of the baseline WVG [9] increases approximately linearly with the word-line count. Therefore, when the baseline WVG is small (i.e., for smaller word-line sizes), the additional fault-tolerant control logic constitutes a larger fraction of the baseline area, resulting in a higher relative overhead. As the number of word lines increases, the baseline WVG area grows nearly linearly, whereas the additional fault-tolerant control logic increases only marginally. Consequently, the relative overhead decreases rapidly for larger arrays.

**Figure 13:**
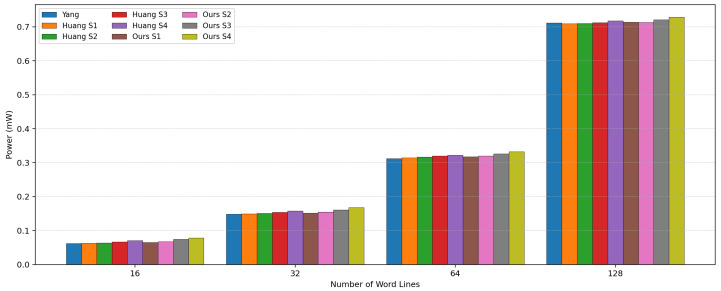
The power consumption of different methods (for the number of word lines from 16 to 128).

**Figure 14:**
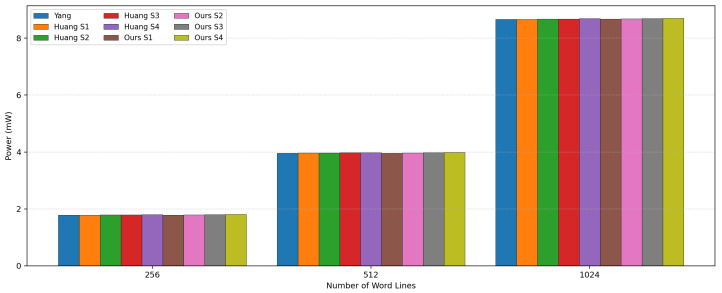
The power consumption of different methods (for the number of word lines from 256 to 1024).

**Figure 15:**
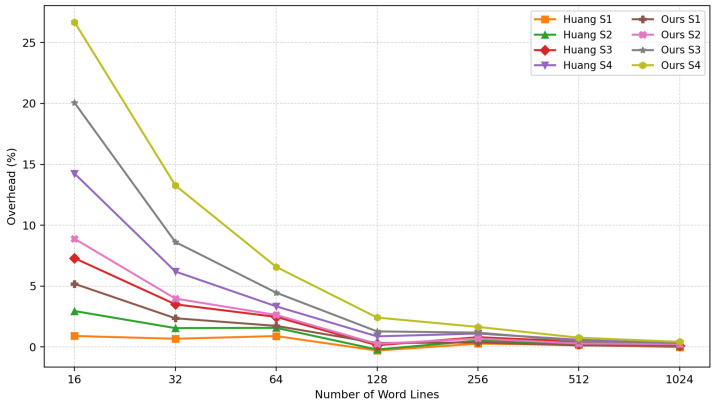
The percentage of power dissipation overhead introduced by the fault-tolerant mechanisms.

For power analysis, Figure 13 and Figure 14 present the results of the power comparison of the three methods under different CIM array sizes. The results show that, compared to the original WVG architecture (i.e., Yang et al. [9]), both the previous fault-tolerant method (i.e., Huang et al. [19]) and the proposed fault-tolerant method (i.e., ours) introduce additional power consumption. Figure 15 illustrates the percentage of additional power consumption introduced by fault-tolerant mechanisms for different numbers of word lines. As the number of word lines increases, the power consumption overhead of the fault-tolerant mechanisms is rapidly amortized.

In Figure 13 and Figure 14, the power consumption of the proposed approach with four spare rows (denoted S4) is the highest among all design configurations. This is because compared with the design of Huang et al. [19], the proposed approach requires additional control logic to support the multi-pass control mechanism. Furthermore, among all configurations of the proposed approach, S4 has slightly more complex control logic than S3, S2 and S1, since it must support remapping for a larger number of spare rows. Consequently, the S4 configuration exhibits the highest power consumption among all evaluated design configurations.

Improving fault tolerance typically involves trade-offs with other performance metrics. Compared to the original WVG [9] and the design of Huang et al. [19], the proposed method supports a larger fault set and can handle both F≤S and F>S cases. This enhanced fault-tolerance capability requires additional fault-aware selection and remapping control logic, leading to increased area and power consumption. Moreover, when F>S, the computation may require multiple passes, causing latency to increase with the number of required passes. Nevertheless, the experimental results show that the area and power overheads are gradually amortized as the number of word lines increases. Therefore, the proposed method trades a moderate hardware overhead and potential latency overhead for improved correctness and reliability under higher fault-density conditions.

This paper focuses on the design of a fault-tolerant WVG. Therefore, the evaluation mainly compares the area and power of different WVG designs. From a whole-CIM-system perspective, the overhead of the WVG is relatively small. The WVG serves only as a peripheral control module for generating word-line activation vectors, whereas a complete CIM macro also includes the memory crossbar array, word-line/bit-line drivers, sensing circuits, ADCs/DACs, shift-and-add circuits, and other peripheral components. Since these components usually dominate the total area and power consumption, the system-level impact of the WVG overhead is expected to be smaller than the normalized WVG-level results, especially for larger CIM arrays. Therefore, the proposed method trades a moderate peripheral-control overhead for improved fault tolerance and reliable CIM operation under faulty-word-line conditions.

We use the CIM macro reported in [36] as an example. The macro contains 128 word lines and occupies an area of 250,000 $\mu m^{2}$ in TSMC 40 nm technology. On the other hand, assuming four spare rows, the WVG area is 10,443.46 $\mu m^{2}$ in Yang et al. [9], 10,738.07 $\mu m^{2}$ in Huang et al. [19], and 10,870.07 $\mu m^{2}$ in the proposed design. Relative to the CIM macro area reported in [36], the area overheads are 4.01%, 4.12%, and 4.17%, respectively. Therefore, from the perspective of the entire CIM macro, the additional overhead introduced by the proposed fault-tolerant WVG is relatively small.

The above results show that, compared to the original WVG architecture [9] and the fault-tolerant method presented by [19], the proposed approach can support a more comprehensive fault-tolerant mechanism while still maintaining a low area and power overhead. This indicates that the proposed multi-pass control mechanism not only preserves the high-efficiency sparse-control capability of the original DOF architecture but also improves its reliability under high-fault-rate environments.

## 6. Conclusions

This work proposes a comprehensive fault-tolerant mechanism to mitigate the impact of faulty word lines on computational correctness and system reliability in RRAM-based CIM architectures. The proposed approach extends the DOF method by incorporating spare-row allocation, vector-correction logic, and prefix-sum correction logic, thereby enabling the system to dynamically bypass faulty word lines during each computation cycle while preserving the correct formation of virtual OUs. Furthermore, when the number of faulty word lines exceeds the available redundancy capacity, a multi-pass computation mechanism is employed to ensure computational correctness and maintain reliable system operation.

Our main contribution is the establishment of a comprehensive fault-tolerant mechanism for the DOF architecture, enabling RRAM-based CIM systems to maintain functional correctness and high reliability under a limited number of spare rows. Compared with previous work, the primary advantage of our approach is its ability to ensure correct computation even when the number of activated faulty rows exceeds the number of available spare rows.

Experimental results demonstrate that the hardware overhead of the proposed fault-tolerant mechanism is approximately constant. That is, when the memory size increases (i.e., as the number of word lines grows), the additional hardware cost introduced by the fault-tolerant mechanism increases only slightly. Consequently, as the memory size becomes larger, the proportion of area and power overhead contributed by the fault-tolerant mechanism relative to the overall DOF circuitry decreases significantly. Therefore, in large-scale RRAM-based CIM systems, the hardware overhead of the proposed fault-tolerant mechanism accounts for only a small fraction of the overall DOF circuit, indicating the high practicality of the proposed approach.

For brevity and simplicity, this paper assumes that all spare rows are fault-free. Nevertheless, the proposed method can be readily extended to handle defective cells in spare rows. In such a scenario, the Mask Vector must additionally encode the fault status of spare rows, while both the prefix-sum computation and Activation Vector generation must also account for defective spare rows. Consequently, the control logic becomes more complex. We leave the investigation of fault-prone spare rows as future work.

## Figures and Tables

**Table 1:** An example for fault-aware multi-pass word line activation.

**Fault Indicator**	0001010010100000	
**Input Vector**	1001011010110110	
**Mask Vector**	1000001000010110	
**Pass 1**		
**Run Vector**	1001011000010110	
**Prefix Sum**	111122444445567723	
**Match** **Vector**	**L = 1, H = 3**	111111000000000010
	**L = 3, H = 5**	000000111110000001
	**L = 5, H = 7**	000000000001110000
	**L = 7, H = 9**	000000000000001100
**Activation** **Vector**	**L = 1, H = 3**	100000000000000010
	**L = 3, H = 5**	000000100000000001
	**L = 5, H = 7**	000000000001010000
	**L = 7, H = 9**	000000000000001000
**Pass 2**		
**Run Vector**	0000000010100000	
**Prefix Sum**	000000000112222212	
**Match Vector**	**L = 1, H = 3**	000000000111111111
**Activation Vector**	**L = 1, H = 3**	000000000000000011

## Data Availability

The data used to support the findings of this study are included in this paper.
